# Ovine fetal swallowing responses to polyhydramnios

**DOI:** 10.1002/phy2.279

**Published:** 2014-03-26

**Authors:** Robert A. Brace, Debra F. Anderson, Cecilia Y. Cheung

**Affiliations:** 1 Division of Maternal Fetal Medicine, Department of Obstetrics and Gynecology, Oregon Health and Science University, Portland, Oregon; 2 Center for Developmental Health, Knight Cardiovascular Institute, Oregon Health and Science University, Portland, Oregon

**Keywords:** Amniotic fluid volume regulation, fetal swallowing, polyhydramnios, sheep fetus

## Abstract

Swallowing of amniotic fluid by late gestation fetuses increases when amniotic fluid volume (AFV) is elevated. Our objectives were to quantitatively characterize fetal swallowing when AFV is elevated above normal to polyhydramniotic levels and to explore the mechanisms that mediate these changes. Late gestation fetal sheep were studied under basal conditions and during intra‐amniotic infusion of lactated Ringer's solution. Control AFV averaged 631 ± 214 mL (SE,* n* = 6), swallowed volume was 299 ± 94 mL/day, and there were 5.7 ± 1.8 bouts/day of rapid swallowing. During intra‐amniotic infusion, AFV (3065 ± 894 mL) and daily swallowed volume (699 ± 148 mL/day) increased (*P* < 0.05) and the number of bouts reached a maximum of 13.7 ± 2.0 bouts/day when AFV exceeded 1500 mL. Unexpectedly, the volume swallowed per bout (57.3 ± 5.8 mL, *n* = 102) did not vary with AFV (*r* = 0.023, *P* = 0.81). Neither the number of swallows/day nor the volume/swallow changed consistently with elevated AFV. Daily swallowed volume increases and reaches a maximum of twice normal as AFV approaches polyhydramniotic levels. Mechanistically, the increase in swallowing was achieved primarily by an increase in the number of bouts of swallowing per day rather than the expected passive increase in volume per bout. This implies changes in fetal behavior as AFV was elevated. Furthermore, swallowed volume was four times more sensitive to increases in AFV than reported previously.

## Abstract (precis)

Daily swallowed volume in the ovine fetus varies sharply with changes in amniotic fluid volume around normal and reaches a maximum as amniotic fluid volume exceeds 2000 mL. These changes are mediated by altering the number of bouts of swallowing per day rather than the volume swallowed per bout. Retrograde esophageal flow was normally low but became large as daily swallowed volume increased above 900 mL/day.

## Introduction

In late gestation, fetuses swallow large volumes of amniotic fluid (AF) daily (Pritchard 1965; Bradley and Mistretta 1973; Mistretta and Bradley 1975; Abramovich et al. 1979; Tomoda et al. 1985). Thus swallowing of AF by the fetus has been accepted as a major contributor to the regulation of amniotic fluid volume (AFV). However, neither the alterations in fetal swallowing as AFV changes nor the mechanisms that mediated these changes have been well characterized.

Fetal swallowing under normal conditions has been most often studied in the chronically catheterized ovine fetus. Although individual swallows occur throughout the day, most of the volume ingested by the fetus results from bouts of rapid swallowing when large volumes are ingested over short periods of time (Bradley and Mistretta 1973; Harding et al. 1984a,b; Sherman et al. 1990). However, it is unknown whether changes in daily swallowed volume are mediated by changes in the number of bouts per day, the volume per bout or a combination of both. Furthermore, in addition to antegrade flow through the esophagus into the stomach, retrograde flow also occurs in both human (Bowie and Clair 1982) and sheep fetuses (Sherman et al. 1990). Currently it is unknown whether retrograde flow correlates with antegrade flow or varies over the full range of possible AFVs.

Few studies have characterized fetal swallowing when AFV is different from normal. In a study of oligohydramnios in sheep, Kullama et al. (1994) found that the daily swallowed volume and the volume per swallow were reduced when AFV was 38% of its initial value. Fetal swallowing also has been reported to be elevated when AFV is above normal. However, most of these studies (Tomoda et al. 1985; Gilbert and Brace 1988; Brace 1989) used an indirect tracer disappearance method for determining swallowed volume. The method not only overestimates swallowing because the tracer rapidly crosses the amniotic membrane and enters fetal blood (Faber and Anderson 2002) but also does not allow the characteristics of swallowing to be determined. Our recent study provided an equation describing fetal swallowing when AFV is above normal (Brace et al. 2013). However, that study included hypoxic fetuses. Because hypoxia suppresses fetal swallowing (Sherman et al. 1991; Brace et al. 1994a), the study does not represent the swallowing responses of nonhypoxic fetuses to increases in AFV. Furthermore, the changes in swallowing characteristics that mediate the increased swallowed volume when AFV is greater than normal have not been reported.

The objective of this study was to quantitatively characterize the changes in individual swallows, bouts of swallowing, and daily swallowed volume in nonhypoxic fetuses when AFV was experimentally elevated to polyhydramniotic levels. In contrast to our previous speculation that passive mechanisms would mediate the increase in daily swallowed volume as AFV increases (Brace et al. 2013), the present results are consistent with the concept that behavioral changes alter daily swallowed volume as AFV varies.

## Materials and Methods

### Ethical approval

These studies were approved by our Institutional Animal Care and Use Committee (IACUC) and we followed the National Research Council's *Guide for the Care and Use of Laboratory Animals* (Institute for Laboratory Animal Research 2010).

### Experimental preparations

Six late gestation singleton ovine fetuses were catheterized at 117–123 days gestation as detailed elsewhere (Thurlow and Brace 2003; Faber et al. 2005; Robertson et al. 2009). Briefly, using inhalation anesthesia and aseptic techniques, the right fetal carotid artery was catheterized for sampling of fetal blood. The urachus was ligated to prevent urine entry into the allantoic sac. A precalibrated flow probe (Transonic Systems, Ithaca, NY) was secured around the mid cervical esophagus for measuring fetal swallowing (Bradley and Mistretta 1973; Sherman et al. 1990; Thurlow and Brace 2003). Additional catheters were attached to the fetal skin to allow access to the AF. One end of a catheter was placed in the trachea and the other end attached to the fetal skin to allow secreted lung liquid to enter the amniotic sac. Normally half of the secreted lung liquid enters the AF with the remainder being swallowed as it exits the trachea (Brace et al. 1994b). The advantage of tracheal catheterization is that all swallowed fluid is AF except for a minor amount of oral‐nasal secretions (Brace 1986).

### Experimental procedures

Experiments began 5–6 days after surgery. The animals were housed in individual pens with a 12 h/12 h light/dark cycle and free access to food and water. Fetuses were randomly assigned to control or intra‐amniotic infusion protocols, each lasting 2 or 3 days. Each fetus completed both protocols. At the beginning of each protocol, AF was drained and replaced with 1 L of lactated Ringer's solution warmed to fetal body temperature so all protocols began with the same volume and composition of AF. At the end of the first protocol, AF was replaced with 1 L of lactated Ringer's solution and the second protocol was initiated. The control protocol consisted of monitoring without infusions. During the infusion protocol, lactated Ringer's solution was infused at a constant rate of 2 or 4 L/day (1.4 or 2.8 mL/min) in order to increase AFV. The differing infusion rates and durations were used to achieve different AFVs. Throughout each protocol, the esophageal flow signal was sampled at 100 Hz and stored on disk. At the end of each protocol, fetal arterial blood was sampled for blood gases and pH (model 725 analyzer; Radiometer, Westlake OH). AFV was measured by drainage at the end of each protocol (Robertson et al. 2009). In previous studies, we have verified at autopsy that drainage removed all AF except for a minor amount contained in fetal wool and have previously shown that drainage AFV values are in agreement with indicator dilution values except for fluid contained in the catheters and fetal wool (Brace and Cheung 2011).

At the end of the study, the animals were euthanized using an IACUC approved intravenous euthanasia solution and membrane integrity verified by the absence of holes or tears in the membranes.

### Calculations

A swallow (antegrade flow) was defined as a positive deviation in the esophageal flow signal by more than 4 SD above baseline noise, lasting more than 0.02 sec, and continuing until returning to the baseline. A regurgitation (retrograde flow) was similarly defined as flow occurring in the opposite direction. Although a swallow is generally considered esophageal passage of fluid into the stomach (rumen) and regurgitation the passage of fluid from the stomach through the esophagus, it was not possible with our flow measurement at the mid cervical esophagus to determine whether the passing fluid was moving into or out of the fetal stomach rather than moving into or out of the thoracic esophagus (discussed below). Thus, the terms swallow and antegrade flow are used interchangeably as are the terms regurgitation and retrograde flow. The daily swallowed volume was calculated by computer integration of both positive and negative deviations from baseline in the esophageal flow signal over 24 h. Zero flow (baseline) was calculated with the computer by continuously adjusting the zero flow value whenever a swallow or regurgitation was not present.

In previous studies, several different definitions of a bout of swallowing have been used based on either fluid movement through the esophagus or EMG activity of the muscles associated with swallowing (Bradley and Mistretta 1973; Mistretta and Bradley 1975; Harding et al. 1984a; Sherman et al. 1990). Because there is no standard definition of a bout and because the focus of this study was the volume swallowed relative to AFV, we arbitrarily used a definition of a bout of swallowing as rapid swallowing of 10 mL or more over 20 min or less as a primary definition of a bout. The lower limit of 10 mL for a bout of swallowing is consistent with the cervical esophageal flow studies of Bradley and Mistretta (1973) and Mistretta and Bradley (1975). The 20 min time period was derived from the visual appearance of graphs of cumulative swallowed volume over 24 h that are included in this study. We also used a secondary definition of a bout as swallowing of 5 mL or more over 5 min or less as calculated from consecutive 5 min periods. Because previous studies have shown that AFV stabilizes within 1–2 days after a volume disturbance (Brace and Cheung 2004), the esophageal flow signal was analyzed for swallowing characteristics over the last 24 h of each 2–3 day protocol.

### Data presentation and statistics

Data are presented as mean ± SE. A paired *t*‐test was used for comparison of values during control conditions versus those during elevated AFV. The volumes of individual swallows were tested for normality of distribution and skewness using chi‐square and gamma tests. Linear and nonlinear least squares regression was used to determine statistical relationships between variables. Nonlinear regression included an exponential function and two interconnected straight lines, the intersection of which is referred to as the breakpoint. A one factor analysis of covariance (ANCOVA) was used to compare regression relationships between an individual swallow volume and swallow duration under control conditions versus augmented AFV. The coefficient of determination was calculated from the correlation coefficient as *r*^2^ × 100%. A *P* value of ≤0.05 was taken as statistically significant.

## Results

### Individual swallows and regurgitations

**Figure 1.**
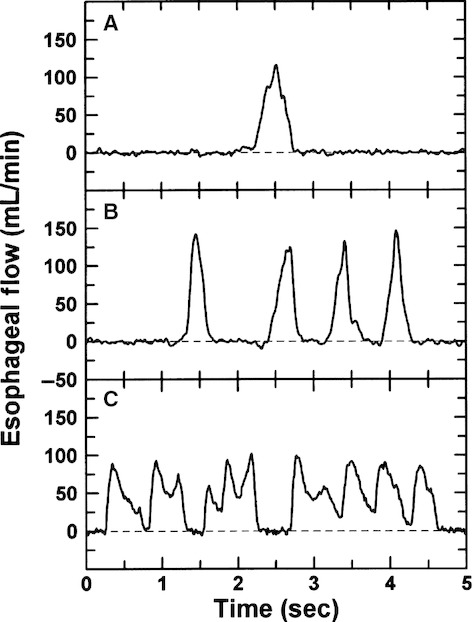
Patterns in fetal swallowing. (A) Isolated swallow (volume = 0.55 mL); (B) train of isolated swallows (volumes = 0.42, 0.44, 0.39, and 0.38 mL); (C) flow during a 5 sec portion of a bout of rapid swallowing (volumes = 0.39, 0.46, 0.66, and 1.73 mL). Dashed lines indicate baseline (zero) flow.

Multiple patterns were present in esophageal flow, including isolated single swallows (Fig. 1A) as well as trains of single swallows (Fig. 1B) and bouts of swallowing (Fig. 1C). Single swallows rarely lasted more than 0.7 sec with volumes usually <0.7 mL. In comparison, cumulative volume typically was 1–3 mL during trains of single swallows. During bouts of swallowing, esophageal flow rate frequently did not return to the baseline prior to the passage of the next bolus of fluid (Fig. 1C).

**Figure 2.**
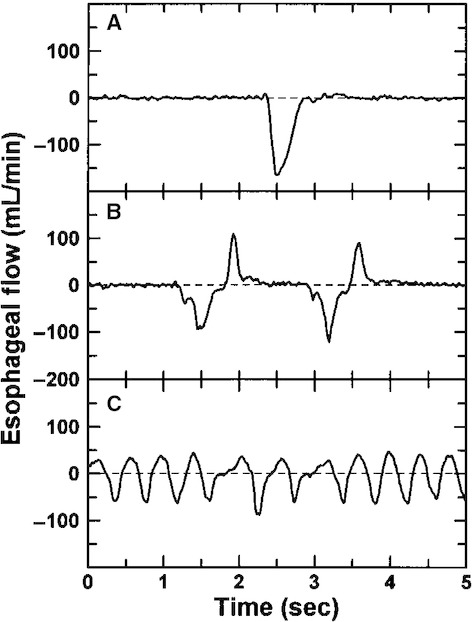
Patterns during retrograde flow. (A) Isolated regurgitation (volume = 0.71 mL); (B) regurgitations followed by swallows (net retrograde flow = 0.19 mL); (C) oscillatory flow (net retrograde flow = 0.33 mL).

An isolated regurgitation largely mirrored an isolated swallow (Fig. 2A). However, trains of retrograde flow often were a series of regurgitations with a swallow after each (Fig. 2B). The opposite pattern of trains of individual swallows followed immediately by retrograde flow was not seen. In addition, unlike net fluid intake during bouts of swallowing, bouts of what might be considered regurgitations rarely occurred but instead typically were high‐frequency back‐and‐forth movements of fluid within the esophagus with little or no net fluid transport (Fig. 2C).

In each fetus, the volume per swallow (*Y* in mL) was positively correlated with duration of the swallow (*X* in sec) and there was no significant difference (ANCOVA, *P* > 0.05) between regression equations for control conditions compared to augmented AFV. The mean regression equation was *Y* = 1.86 (±0.15) × *X* − 0.18 (±0.03). Regurgitations behaved similarly, with the mean regression equation *Y* = 1.38 (±0.19) × *X* − 0.09 (±0.02). When AFV was expanded, although the average volume/swallow increased significantly in five of six fetuses, the change in mean volume/swallow was not significant when all six animals were compared (0.35 ± 0.05 mL vs. 0.23 ± 0.05 mL, *n* = 6, *P* = 0.19). Similarly, the number of swallows/day increased in four of six fetuses when AFV was increased but the mean values for all fetuses during control versus expanded AFV conditions were not significantly different (2638 ± 1347 swallows/day vs. 2280 ± 726 swallows/day, *n* = 6, *P* = 0.68). The volume per regurgitation increased when AFV was elevated in five of six fetuses and there was no consistent change in the number of regurgitations per day. In each fetus, the distribution of the volume/swallow was significantly non‐normal, with skewing to the right due to existence of swallows with high volume.

### Bouts of swallowing and regurgitation

**Figure 3.**
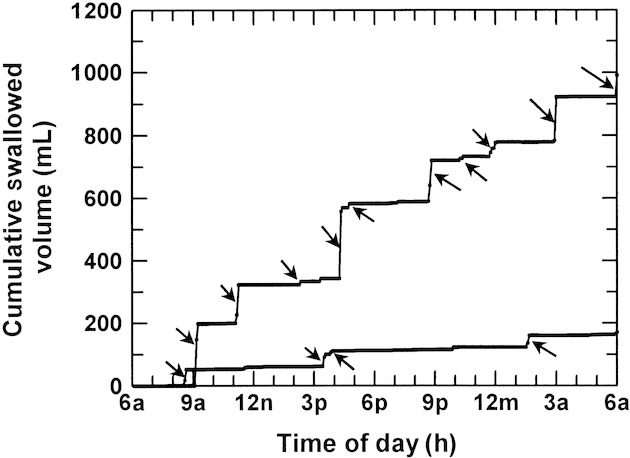
Cumulative swallowed volume/24 h in one late gestation ovine fetus calculated from 5 min swallowed volumes during control conditions (lower line, amniotic fluid volume = 350 mL) and during expansion of amniotic fluid volume (upper line, amniotic fluid volume = 1960 mL). Arrows show individual bouts of rapid swallowing defined as swallowing 10 mL or move over 20 min or less.

**Figure 4.**
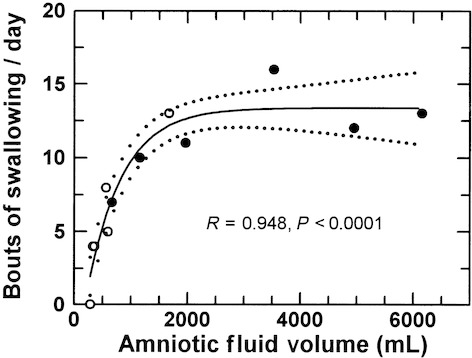
Bouts of swallowing/day during control conditions (open circles) and when amniotic fluid volume was increased by intra‐amniotic fluid infusion (filled circles). A bout of swallowing was defined as swallowing 10 mL or more over 20 min. Data fit with an exponential function: *Y* = *A* + *B* × 10^(*C* × *X*); *A* = 13.36 (plateau value), *B* = −18.0, *C* = −0.000706.

Graphs of the cumulative swallowed volume over 24 h allowed visualization of bouts of swallowing over time (Fig. 3). In each fetus, using our primary definition of a bout of swallowing (10 mL or move over 20 min), the number of bouts/day of rapid swallowing increased when AFV was elevated compared to control AFVs (11.5 ±1.2 bouts/day vs. 5.7 ± 1.8 bouts/day, *P* = 0.0088). Using our secondary definition of a bout of swallowing (5 mL or more over 5 min), the changes were similar in that there were approximately twice as many bouts/day when AFV was elevated compared to control AFV (22.5 ± 3.7 bouts/day vs. 12.0 ± 3.8 bouts/day, *P* = 0.0008). Because of the similarity of the results for these two definitions of a bout of swallowing, subsequent data will use our primary definition of a bout. The number of bouts/day was nonlinearly related to AFV (*r* = 0.95, Fig. 4), reaching a maximum of 13.7 ± 2.0 per day when AFV exceeded 1500 mL.

**Figure 5.**
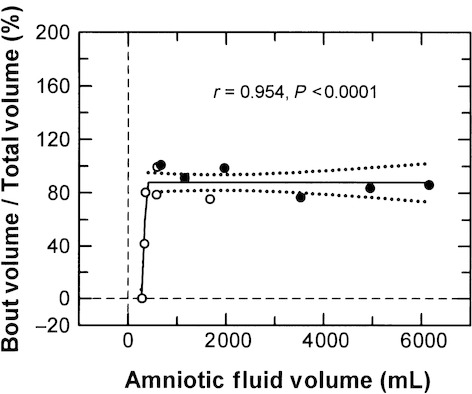
The relative contribution of bouts of swallowing to the total volume swallowed/day as a function of amniotic fluid volume. A bout of swallowing was defined as swallowing 10 mL or more over 20 min. Open circles, control conditions; filled circles, elevated amniotic fluid volume. Solid line, regression line; dotted lines, 95% confidence interval about the regression line. Breakpoint = 410 mL.

Under control conditions, 62.6 ± 14.6% of the daily swallowed volume occurred during bouts of swallowing compared to 89.5 ± 3.8% when AFV was elevated (*P* = 0.18). For the combined data, the percentage of the total daily swallowing that occurred during bouts of swallowing was constant (87.1 ± 3.1%) over the full range of AFVs except when AFVs were <400 mL (Fig. 5). The swallowed volume per bout ranged from 10.2 to 271.4 mL (mean = 57.3 ± 5.8 mL, *n* = 102) and was not correlated with AFV (*r* = 0.023, *P* = 0.81).

On average under control conditions, there was less than one bout of regurgitation per fetus over each 24 h period that resulted in 10 mL or more of fluid moving outward from the fetus. The number of bouts of regurgitations tended to increase but did not change significantly in all fetuses when AFV was expanded.

### Daily swallowed volume, regurgitated volume, and AFV

**Figure 6.**
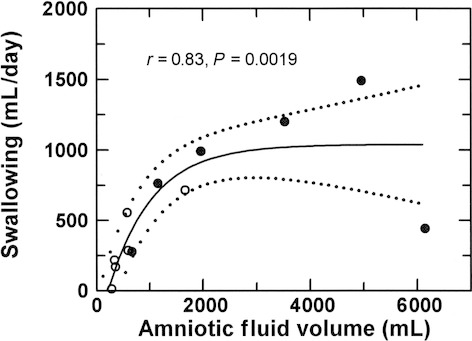
Daily swallowed volume as a function of amniotic fluid volume. Open circles, control conditions; filled circles, elevated amniotic fluid volume. Solid line, regression line; dotted lines, 95% confidence interval about the regression line. Regression equation *Y* = *A* + *B* × 10^(*C* × *X*), *A* = 1039, *B* = −1343, *C* = −0.000525.

There was a nonlinear relationship between daily swallowed volume and AFV, with swallowing at a maximum when AFV exceeded approximately 2000 mL (Fig. 6). There was a 69% (0.83^2^ × 100%) correspondence between the two variables. Swallowed volume (*Y* in mL) also was linearly related to the number of bouts per day (*X*): *Y* = 77.1 × *X* − 68, *r* = 0.81, *r*^2^ = 0.66, *P* = 0.0016. During control conditions, mean swallowed volume was 299 ± 94 mL/day and AFV averaged 631 ± 214 mL. During intra‐amniotic infusion, daily swallowed volume (699 ± 148 mL/day) and AFV (3065 ± 894 mL) increased significantly compared to control values (*P* < 0.05).

**Figure 7.**
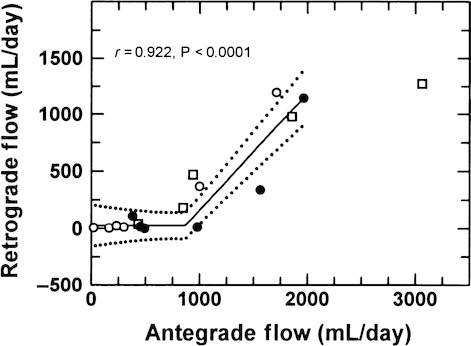
Retrograde flow in the fetal cervical esophagus as a function of antegrade flow. Open circles, control conditions; filled circles, elevated amniotic fluid volume. Solid line, regression line; dotted lines, 95% confidence interval about the regression line. Breakpoint is at antegrade flow = 870 mL/day. The five open squares are thoracic esophageal values calculated from table 1 of Sherman et al. (1990) and were not included in the regression analysis.

When comparing retrograde and antegrade flows, little volume was regurgitated when antegrade flow was <1000 mL/day. However, when antegrade flow exceeded 1000 mL/day, retrograde flow increased in direct proportion with the result that net swallowing did not increase as antegrade flow exceeded 1000 mL/day (Fig. 7).

### Blood gases and pH

At the end of the control period, fetal arterial pH was 7.358 ± 0.010, carbon dioxide tension was 51.9 ±0.9 mmHg, and oxygen tension was 21.3 ± 0.7 mmHg. These values were unchanged at the end of the infusion (paired *t*‐test), averaging 7.362 ± 0.01, 50.7 ± 0.7, and 22.0 ± 0.8 mmHg, respectively.

## Discussion

There are several novel findings in this study. First, this study suggests that fetal swallowing may make a more important contribution to the regulation of amniotic fluid volume than previously recognized. With data from different laboratories under difference experimental conditions including hypoxia (Brace et al. 2013), fetal swallowing changed by 0.3 mL/day for each 1 mL increase in AFV around a normal volume of 600–800 mL. In this study with nonhypoxic fetuses, swallowing changed by 1.1 mL/day for each 1 mL deviation in AFV around normal. Thus, fetal swallowing is approximately four times more sensitive to changes in AFV than previously suggested. This implies that, in the normal, nonhypoxic fetus, swallowing provides an important protective mechanism against polyhydramnios because volume‐dependent alterations in swallowing tend to minimize the changes in AFV that would otherwise occur.

Second, although previous studies have reported fetus‐to‐fetus variations in the number of daily bouts of rapid swallowing (Bradley and Mistretta 1973; Mistretta and Bradley 1975; Harding et al. 1984a; Sherman et al. 1990), the observation that the number of daily bouts of fetal swallowing varies as a function of AFV is new. This relationship is so strong that a majority (66%) of the AFV‐dependent changes in daily swallowed volume can be explained by changes in the number of bouts per day. This conclusion is independent of our definition of a bout of swallowing because, using either of our two definitions, the number of daily bouts of rapid swallowing doubled when AFV was elevated compared to control conditions. Definitions aside, the observation is important because, with bouts of swallowing mediated by central nervous system mechanisms (Sherman et al. 1990), it appears that the fetal brain responds to differentially stimulate fetal swallowing over a broad range of AFVs through unknown mechanisms. It might be suggested that changes in swallowed fluid composition and osmolality induced by the infusion mediate the increase in number of bouts through changes in taste perception (Bradley and Mistretta 1973; Mistretta and Bradley 1975). This appears unlikely as previous studies found only small or no changes in AF composition during similar intra‐amniotic infusions (Brace and Cheung 2004; Robertson et al. 2009). Furthermore, as shown in Figure 6, the swallowed volume for any given AFV was the same in infused and noninfused fetuses over the range of AFVs of approximately 500–1500 mL. These observations argue against AF compositional effects on swallowing in this study.

Third, the increase in swallowing with elevated AFV does not appear to be due to passive mechanisms as previously speculated (Brace et al. 2013). If the mechanism was passive, then the volume swallowed per bout would increase as AFV was elevated because more fluid is available. However, with no increase in the volume swallowed per bout as AFV increased (*r* = 0.023), it is clear that nonpassive mechanisms were the primary determinant of the fetal swallowing responses. Although changes in compression of the fetus by the uterine wall occurs as AFV varies (Shields and Brace 1994; Brace et al. 2013), there are no known mechanisms by which alterations in uterine compression would explain the changes in the number of bouts of swallowing/day as AFV deviates from normal while the volume swallowed per bout is unchanged.

Fourth, the fraction of the daily swallowed volume that occurs during bouts was constant at 87% as AFV varied except for the lowest AFVs. Although there are no previous studies exploring changes in the fractional volume as a function of AFV for comparison, this observation is noteworthy because it shows that, as the volume swallowed during bouts increased, the volume swallowed in the absence of bouts (13%) also increased. In five of six fetuses that increased their volume per swallow, this 13% appears to be a passive contribution to the increase in daily swallowed volume. This may be related to an increase in the ability of the fetus to open its mouth and ingest fluid as AFV expands because compression by the uterus would be reduced (Shields and Brace 1994).

In this study under control conditions, the volume of regurgitated fluid in a majority of the fetuses was very small, increasing only when antegrade flow exceeded 1000 mL/day. This finding may appear to contrast with the previous observation in late gestation ovine fetuses that retrograde flow averaged 23% of antegrade flow (Sherman et al. 1990). However, a comparison of retrograde and antegrade flows in the thoracic esophagus (open squares in Fig. 7) calculated from table 1 of Sherman et al. (1990) with our mid cervical esophageal flow data shows very close agreement except for one outlier with the highest antegrade flow from their study. Thus, the two studies are in agreement even though the mean daily swallowed volumes differed. The reasons for the latter are unknown.

In this study, the volume of fluid passing through the cervical esophagus per swallow averaged approximately 0.25 mL under control conditions and 0.35 mL when AFV was expanded. This is much less than the value of 0.9 ± 0.1 mL (mean ± SE) for the thoracic esophagus as reported by Sherman et al. (1990) as we rarely observed volumes at or above 0.9 mL/swallow. This difference may reside at least in part in the structural/functional differences between the cervical esophagus of this study versus the thoracic esophagus in the report by Sherman et al. (1990) in that the cervical esophagus is usually collapsed whereas the thoracic esophagus acts as a fluid reservoir (Sherman et al. 1990). Similar differences between thoracic and cervical esophagi occur in human fetuses (Brugger et al. 2011).

## Acknowledgments

We thank Sonnet Jonker, Samantha Louey, Robert Webber, and Loni Sochi for their assistance with these studies.

## Conflicts of Interest

None declared.
